# 

**Published:** 1914-11-15

**Authors:** 


### ANNOUNCEMENTS.

### Relief Insurance.

#### HELP FOR ALL.

Have you ever considered who will look after your family when you can not support them? Here is your opportunity. Sign the enclosed blank and send it along. A suggestion has been offered that what you estimate your time worth for one hour shall be your annual fee to the Relief Insurance.

You may say that is all well and good, but I do not require insurance. Just stop. Do you remember any schoolmate, teacher or dental friend that did not require any aid when in his old age he could not support himself? There are over 500 today in the United States, and five years ago they were equally as independent or more so than you are to-day.

Do not hesitate, for the old adage "He who hesitates is lost," may prove that you were leaving undone that which should be a comfort to you and yours.

Your subscription may be sent to any one of the following committee:

Dr. L. G. Noel, 527½ Church St., Nashville, Tenn.

Dr. E. S. Gaylord, 63 Trumbull St., New Haven, Conn.

Dr. Wm. T. Chambers, 504 California Bldg., Denver, Colo.

Dr. Jas. McManus, 80 Pratt St., Hartford, Conn.

---

NATIONAL DENTAL ASSOCIATION.

Relief Insurance Fund Committee.

Member............State Dental Association.

I hereby subscribe ($......)annually as long as I am financially able to the Relief Insurance Fund of the National Dental Association.

Date....................

Signed........................

Address..........................

### NOTICE.

MEETING OF THE NATIONAL ASSOCIATION OF DENTAL FACULTIES

The National Association of Dental Faculties will hold its meeting on the twenty-sixth and twenty-seventh of January at Ann Arbor, Michigan. Headquarters, The Allenel Hotel.

This meeting will precede the Teachers Association meeting which will be held from the twenty-eighth to the thirtieth. Besides the regular business there will be several papers of interest to educators read before the Association.

The Executive committee meets at nine o'clock Tuedsay the twenty-sixth. Regular session will open at ten.

B. Holly Smith,
Chairman of Executive Committee.
Charles Channing Allen,
Secretary.

OHIO STATE DENTAL SOCIETY.—The forty-ninth annual session of the Ohio State Dental Society will be held in Memorial Hall, Columbus, Dec. 1, 2 and 3, 1914.

Papers will be read by Dr. Wm. A. Giffen of Detroit on "Technic for Taking Impressions and Making Models for Constructing Artificial Dentures", giving demonstrations with moving pictures; Dr. H. W. McMillan of Cincinnati, on "Diagnosis and Treatment of Tri-facial Neuralgia"; Dr. W. W. Curtiss, Greenfield, O., on Conservation vs. Radicalism"; Dr. J. R. Callahan of Cincinnati, "A Lanturn Lecture on the Use of Rosin in Operative Dentistry".

A selected list of progressive clinics will be given on Wednesday forenoon and general clinics on Thursday forenoon.

One evening will be given to a Health Conservation Conference to be participated in by all professions and interests devoted to the furtherance of human health and welfare.

A cordial invitation is extended to all society members from other states.

F. R. Chapman, Sec'y.

AMERICAN ACADEMY OF ORAL PROPHYLAXIS. The American Academy of Oral Prophylaxis and Periodontology will meet in Washington City at the Willard Hotel, November, 5, 6, and 7, 1914.

DEDICATION FORSYTH INFIRMARY.—I take this means of advising you relative to the dedication of the Forsyth Dental Infirmary for Children, Boston, as I feel sure that this notice will prove of interest to some of the readers of your "Dental Register."

The dedication is to take place on November 24th, and we wish to extend a cordial invitation to all the members of the dental profession to be present.

There will be many new and interesting features relative to dental equipment to be seen, and I am sure that those accepting our invitation will be well repaid. It will also give them an opportunity to see the magnititude of our undertaking, and something of the inner workings of the institution.

Trusting that you will be able to print notice of this dedication, and with kind regards, I am

Very truly yours,

HAROLD DE. W. CROSS,

Director.

### INDIANA STATE BOARD OF DENTAL EXAMINERS.

The next meeting of the Indiana State Board of Dental Examiners will be held at the State House, Indianapolis, commencing Monday, November 16th, and continuing five days. For application blank and full particulars address

FRED. J. PROW, D.D.S., Secretary,

Bloomington, Indiana.

### THE WEST VIRGINIA STATE DENTAL SOCIETY.

The West Virginia State Dental Society elected following officers:

President, W. B. Conoway, Clarksburg; first Vice-President, Homer Mannon, Huntington; second Vice-President, J. S. Stone, Clarksburg; Secretary, Dr. Parsons, Huntington; Treasurer, C. C. Clark, Blacksville.

### THE PANAMA PACIFIC DENTAL CONGRESS.

The Committee of Organization of the Panama Pacific Dental Congress wishes it distinctly understood that the Panama Pacific International Exposition will not be postponed, but will open on time, as will also the Panama Pacific Dental Congress. The authority for this statement is impressed in the following letter from President Moore of the Exposition Company:—

TO THE COMMISSIONERS FROM FOREIGN NATIONS AND FROM THE STATES AND TERRITORIES OF THE UNITED STATES TO THE PANAMA PACIFIC INTERNATIONAL EXPOSITION.

Gentlemen:—

There have been reports that the Exposition, because of the war in Europe, would be postponed. It will not be postponed.

There have been published statements that the war in Europe would seriously affect the commercial or educational importance or the financial success of the Exposition. They will not be so affected.

The Exposition will open on its scheduled date—February 20th, 1915. It will be completely ready when open. It is more than ninety percent completed to-day. Nothing will be permitted to interfere with the consumation of the plans originally laid down.

Many friends and parties in interest have presented arguments in support of postponement for a year. These have been given anxious study and careful analysis. Most of them are merely counsels of timidity, based on nothing save a general feeling of doubt and uncertainty. These are sufficiently answered by saying that there is no longer any doubt or uncertainty as to the success of the Exposition whatever the situation in Europe may be. Other arguments for postponement have some practical foundation, but for every one of these there is a stronger and better argument for proceeding with our plans.

The Exposition will therefore open as scheduled. There is not the slightest reason to believe its success, in any phase, will be any less than that which was so certain before European war broke out. Not one of the nations at war have notified us of an intention to withdraw her participation; France and Italy have in fact notified us that their plans remain unchanged, but even if we should lose the others the interest and importance of the Exposition would still as a whole, surpass all precedent.

As to the Domestic participation, the effect of the

European war seems likely to rather be advantageous than otherwise. The stimulus on exhibits is already felt, as American manufacturers become impressed with the opportunity given by the Exposition for bringing their goods to the attention of the large distributors of Central-South America, the Orient and Canada.

As to attendance, all expert opinion agrees that there is nothing in the situation, even if continued through 1915, that will affect seriously the willingness or ability of the people of the western hemisphere and of the far east to visit the Exposition. Some opinion is firm that travel to California may even be increased by the war. The decision of the Exposition management, has, however, been reached without regard to that consideration. We consider it our duty alike to our Nation, to the participating nations, to our exhibitors and to ourselves to carry out the plans as originally laid down and which, now nearly at fruition, promise the most important, the most beautiful and most successful Exposition in History.

(Signed) CHAS. C. MOORE,
President.

THE PANAMA PACIFIC DENTAL CONGRESS. At the present time matters relating to the Panama Pacific Dental Congress are most encouraging. The attendance so far as can be judged by those who attended the meeting of the National Dental Association at Rochester, and the meeting of the New Jersey State Dental Society, will break all previous records. Everyone said he was coming, and the Committee of Organization wishes to assure all prospective members that they will be well cared for in every way.

Papers and Clinics by some of the most noted members of the dental profession have already been contributed to the program. A large portion of the available exhibit space has been taken by manufacturers and dealers, and the success of this part of the Congress is positively assured.

The new Municipal Auditorium in which the Congress will be held is being rapidly completed, and will afford facilities for this Congress such as no other similar meeting has enjoyed. It is central y located, and may be reached by numerous street car lines from the Exposition Grounds, or any Hotel in San Francisco, in from five to twenty minutes.

The San Francisco Hotel Bureau, with offices in the Flannery Bldg., San Francisco, will look after the reservations for hotel accommodations for our guests. We earnestly advise all those who intend to attend the Congress to make their reservations early. Many Congresses will be held in San Francisco during the months of August and September, 1915, and many will be in session at the time of the meeting of the Panama Pacific Dental Congress, and for obvious reasons the reservation of rooms should not be delayed.

Following is a circular letter from the San Francisco Hotel Bureau:—

Hotel Reservations For 1915—You can get all the rooms you want in San Francisco in 1915 and at reasonable rates.

By official tabulation there are 1328 hotels and rooming-houses, which with 600 apartment-houses represents a total of over 90,000 rooms with accommodations for over 200,000 guests at any one time. In process of construction there are over 150 hotels and apartment-houses that will be completed before the exposition opens giving nearly 15,000 rooms more and in addition there are thousands of flats, and rooms obtainable in private residences. Fully 98 percent of these hotels and apartment-houses have been built within the last 6 years so that all are modern and up-to-date.

With the purpose of making it as easy as possible for prospective visitors to secure any accommodations desired and at guaranteed rates, the San Francisco Hotel Association representing more than 350 hotels, rooming and apartmenthouses, has incorporated the San Francisco Hotel Bureau with a capital of $100,000. This Bureau will make contracts for hotel and other accommodations in any number required during the Exposition year, and it guarantees all its contracts. A San Francisco Bank is its depository. These accommodations will be contracted at rates of from $1.00 to $3.00 a day, per person, European plan, and from $3.50 and up a person, per day, American plan, for any date and for any length of time desired. No fees or charges of any kind will be required, but a satisfactory guarantee must be given that the rooms will be used at the time stated in the contract.

The Bureau has the endorsement of and is operated in conjunction with the Panama Pacific International Exposition; the San Francisco Convention League; the San Francisco Hotel Association and other civic bodies.

Applications for reservations should be directed to Kirk Harris, Manager San Francisco Hotel Bureau, Kearny and Market Sts., San Francisco.

Make your reservations now.

### MEMORIAL RESOLUTIONS ADOPTED BY THE INDIANAPOLIS DENTAL SOCIETY.

Whereas:—

The Great Creator of the Universe has seen fit to take from us our beloved and honored friend and brother, George Edwin Hunt, and

Whereas:—

His labors in his chosen profession have been of lasting and unmeasured benefit to us individually and to the entire profession at large, and

Whereas:—

His services to this society were of the highest order and his kindly council shall be greatly missed and a great loss thereby sustained, therefore

Be It Resolved:—

That the Indianapolis Dental Society does hereby express its great appreciation for all of his many services and its deep grief at his untimely death. Be it further

Resolved:—

That copies of this resoultion be sent to his bereaved wife and daughter and spread upon the minutes of this society and sent to the Indiana Dental Association Bulletin for publication.

By order of the Committee of the Indianapolis Dental Society.

CARL D. LUCAS, CHAIRMAN,
F. R. HENSHAW,
D. A. HOUSE,
T. S. HACKER,
W. E. KENNEDY.

Sept. 22, 1914.

We regret to learn that Dr. W. G. Ebersole, the noted promoter of the National Mouth Hygiene movement, has undergone a serious surgical operation. His friends hope for his speedy recovery.

